# Cyclic Buckling Characterization of an Individual MWCNT Using Quantitative In Situ TEM Axial Compression

**DOI:** 10.3390/nano13020301

**Published:** 2023-01-11

**Authors:** Raz Samira, Adam Cohen, Fernando Patolsky, Noa Lachman

**Affiliations:** 1 Department of Materials Science and Engineering, The Iby and Aladar Fleischman Faculty of Engineering, Tel Aviv University, Tel Aviv 69978, Israel; 2 School of Chemistry, Faculty of Exact Sciences, Tel Aviv University, Tel Aviv 69978, Israel

**Keywords:** carbon nanotubes, in situ deformation, transmission electron microscope, nanoindentation, nanomechanics, buckling, durability

## Abstract

Carbon nanotubes (CNTs) are extremely conductive and flexible, making them ideal for applications such as flexible electronics and nanoelectromechanical systems. However, in order to properly apply them in such devices, their long-term durability must be assessed. In the present study, we demonstrate cyclic loading of a thick MWCNT (175 nm) under axial compression, observed in situ under a transmission electron microscope (TEM). The force was applied via controlled displacement, while real-time TEM videos of the deformation process were gathered to produce the morphological data. The in situ observations combined with force–displacement curves revealed the onset of buckling instabilities, and the elastic limits of the tube were assessed. The MWCNT retained its original structure even after 68 loading–unloading cycles, despite observed clues for structural distortions. The stiffness of the tube, calculated after each loading cycle, was in a 0.15 to 0.28 TPa range—comparable to the literature, which further validates the measurement set-up. These in situ tests demonstrate the resilience of CNTs to fatigue which can be correlated with the CNTs’ structure. Such correlations can help tailoring CNTs’ properties to specific applications.

## 1. Introduction

With the ongoing miniaturization of technology, carbon nanotubes (CNTs) have emerged as promising candidates in a wide range of applications, especially within the field of mechanical electronics [1,2,3]. Among their many properties, CNT excel in high carrier mobility, conductivity, mechanical flexibility, and low weight [4,5,6,7]. These properties, together with their low production cost, make them appealing for flexible electronics, nanotube radios [8], nanotweezers [9,10], and high-frequency nanoelectromechanical systems (NEMS), such as nanoswitches [11], nanorelays [12,13] and nanoresonators [14,15].

The high in-plane stiffness of CNTs is derived from the rigidity of the C–C bonds, and the tensile Young’s modulus can reach even 1 TPa [6,7]. Under compression, however, the mechanical stiffness of the tube can only hold the structure upright until it reaches a point of instability, where the bending stiffness decreases dramatically and the tube geometry changes abruptly. This phenomenon is named Euler buckling, and was previously demonstrated in the literature for both single-wall carbon nanotubes (SWCNTs) and multi-wall carbon nanotubes (MWCNTs) [16,17,18,19,20]. The consensus is that MWCNTs, with their multi-layer structure allowing for more efficient stress distribution, are more suitable for applications requiring resistance to compressive stresses [18,21]. According to Euler’s column formula, *P_critical_ = π*^2^*EI/*(*LK*)^2^ (where *E* is the Young’s modulus, *I* is the moment of inertia—proportional to the radius squared, *L* is the length of the column, and *K* is the boundary condition coefficient), the critical load for buckling *P_critical_* sustainable by thicker CNTs, with a larger moment of inertia, is higher [22].

Another phenomenon unique to MWCNTs under compression is the rippling effect, which is manifested in the appearance of a wave-like distortion in the inner arc of a bent nanotube, and in a significant reduction in the bending stiffness [23,24,25,26]. Rippled and buckled phases also result in a decrease in electrical conductivity [27,28]. Therefore, the durability and flexibility of CNTs against mechanical compression are the primary criteria to evaluate the performance of CNT-based nanoelectromechanical devices and their lifetime reliability.

Although rippling and buckling behavior of CNTs have been extensively modeled [24,29,30,31,32,33,34], experimental measurements remain very challenging due to the small dimensions, thus characterizations are incomplete [18]. An effective method to evaluate the nonlinear response of a single CNT against compression is to conduct quantitative mechanical testing using in situ transmission electron microscope (TEM). The greatest advantage of the in situ technique is that the entire operation can be seen live, while mechanical data is collected simultaneously. By correlating the load–displacement (F–D) data to the onset of buckling and rippling, one can obtain useful insights into the compression-induced instabilities of CNTs. Studies conducted in the last couple of decades investigated buckling and fracture modes of MWCNTs in TEM, employing an AFM apparatus [19,20,25,35] or a piezoelectrically driven nanoindenter [16,17]. These experiments revealed that CNTs exhibit reversible deformation in response to repeated compression [16,17,20] and that the buckling mode is dependent on their aspect ratio [19,25]. Furthermore, they reported large variations in the Young’s modulus values for different types of MWCNTs, ranging from 0.2 to 1.075 TPa [16,17,19,20,25,35,36,37]. It has been shown that the mechanical properties of CNTs are highly affected by geometry (e.g., diameter, length, alignment), crystalline structure, and concentration of defects [36,37]. Such factors, in turn, depend on the chosen growth method. For example, the arc-discharge method produces highly crystalline CNTs with a modulus of 1 TPa, compared to 0.1–0.4 TPa for chemical vapor deposition (CVD)-grown CNTs [25]. Another example is the bamboo-like CNT (bCNT), whose structure differs from that of regular CNTs by containing separate hollow compartments and bamboo knots that grow along its axis. bCNTs can be grown using different synthesis methods (see review [38]) and their structure typically contains high defect densities, resulting in a modulus smaller than 0.2 TPa [35]. One strategy to improve the quality of MWCNTs produced using CVD processes is via a post-growth treatment such as thermal annealing [39]. Heat treatment in an inert environment at 2200 to 2800 °C eliminates defects in the microstructure, improving the bending modulus to 1 TPa.

In order to assess and possibly even enhance the performance of nanoelectromechanical devices using CNTs, long-term mechanical durability must also be evaluated. Some studies have investigated the durability to compression of an individual thin MWCNT (outer diameters ranging from 13 to 38 nm [16,17,19,20,25,35]). However, durability to compression over a large number of loading cycles has not been assessed. In addition, there is a lack of studies on thick MWCNTs (greater than 100 nm).

In this study, we present extensive compression cyclic loading through an in situ TEM method, in order to evaluate the mechanical durability and flexibility of an individual thick bamboo-like MWCNT (outer diameter of 175 nm). We analyze the compression-induced buckling instabilities, and we elucidate the morphological and structural distortions that might lead to failure. This is a crucial requirement for flexible electronic devices and NEMS, as they need to maintain their performance over multiple iterations.

## 2. Materials and Methods

**Figure 1:**
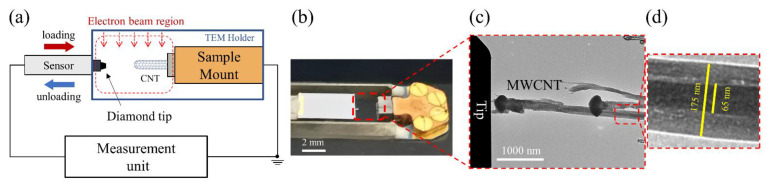
(**a**) Schematic illustration of the experimental setup for in situ TEM nanoindentation; (**b**) photograph of the TEM holder, showing the indenter facing the sample. The position of the indenter tip is controlled by the 3-axis movement of a piezo-motor; (**c**) a TEM image showing the initial position of the diamond tip and a single MWCNT in a compression experiment; (**d**) a magnified image of the CNT measuring the outer diameter of 175 nm and inner diameter of 65 nm, corresponding to 161 walls.

A reoccurring problem in common experimental setups for individual CNT compression [16,19,20,35] is that the tubes are not, in fact, straight nor aligned on the substrate, which leads to bending rather than compression. Furthermore, the CNTs are often deposited on a substrate, rather than grown, resulting in poor adhesion. Herein, plasma-enhanced chemical vapor deposition (PECVD) was used to grow straight and vertically aligned MWCNTs (VACNTs) directly onto a special-purpose substrate, exhibiting great adhesion. The wedge substrate consisted of a long and tall ridge geometry, on which thin films can be deposited and nanoparticles can be grown. The VACNTs were grown on a silicon-wedge substrate using DC/RF PECVD (Black Magic 2, Aixtron, Germany) [40], at 700 °C using a nickel catalyst (3 nm) and C_2_H_2_:NH_3_ 20:80 sccm feedstock, for 1 h (the full process of preparing the wedge and growing the VACNT can be found in the Supplementary Materials). In this synthesis, ammonia is used as a hydrogen-rich reducing agent [41]. The PECVD growth method was chosen, as the electric field in the plasma aligns the nanotubes as they grow, resulting in straight, vertical tubes—crucial for an accurate mechanical compression measurement. In addition, the fabrication process does not require focused ion beam (FIB), thus protecting the material from gallium ion irradiation damage, enabling analysis of their intrinsic mechanical behavior [42,43]. The substrate was fixed on the TEM holder, perpendicular to the electron beam (Figure 1a).

The in situ nanomechanical test used a flat nanoindenter to compress an individual MWCNT and displayed the process of deformation in the TEM simultaneously (Figure 1). The experiments were performed in an FEI Tencai 20 TEM (Hillsboro, OR, USA) with a Bruker (Hysitron, Minneapolis, MN, USA) PicoIndenter 95 (PI-95) TEM holder. The TEM was operated at 200 keV with a field-emission electron source, in bright-field mode. Medium magnification was utilized to have a complete view of the tube and to reduce the radiation and knockout damage. The PicoIndenter used a 3D piezoelectric drive which allowed for precise positioning inside the TEM perpendicular to the target MWCNT (Figure 1c). Important to note is that the tube was slightly tilted, but occupied the same plane as the loading axis as evidenced by both occupying the same focal plane. Additionally, the adjacent tubes (Figure 1c) did not participate in the loading process as they were in a different z-plane to the targeted tube (slightly under-focused, as can be seen by the fringes around it). 

The force was applied along the axis of the tube, in displacement-controlled mode, at a displacement rate of 5 nm/s, and included 200 datapoints per second of force and normal displacement. Videos were recorded using digital capture of a Gatan One View camera (Gatan Inc., Pleasanton, CA, USA) at 10 frames per second and 4K resolution. The load–displacement data and the real-time videos were recorded and synchronized using the frame grabber feature of TriboScan software (Hysitron, Minneapolis, MN, USA). The measured displacement was validated using synchronized imaging. The mechanical properties of the MWCNT were studied using repeated deformation cycles in compression, and individual compression tests, each with an increased maximum displacement to assess the limits of the tubes’ flexibility. The critical force for buckling (*P_cr_*) was determined from the F–D curves by calculating the crossing point between two different slopes of the linear fits in the pre- and post-buckling regions, using OriginLab software (Origin Pro 2016, OriginLab Corporation, Northampton, MA, USA). The pre-buckling region was identified by a linear rise in force and the post-buckling region was identified by the slope change indicating softening transition. The error range of the *P_cr_* values was drawn from the linear-fit calculations, as given by Origin. The *P_cr_* values were further validated using the synchronized video of the F–D curve and the real-time imaging, by identifying the point at which lateral deflection began (see supplementary Figure S5). The *P_cr_* of each cycle was then put in Euler’s column formula to calculate the Young’s modulus. Based on error propagation of *P_cr_* errors, the error range of the modulus was calculated for each cycle. After eight initial cycles, the tube underwent further cyclic loading, compressing first to predetermined maximum displacement and then retracting each time to half its value. In total, 68 cycles of loading and unloading were carried out on a single tube. Post-fracture TEM images were taken for further analysis of the resulting crack. 

## 3. Results and Discussion

The chosen tube had an outer diameter of 175 nm and an inner diameter of 65 nm, which corresponds to a wall thickness of 55 nm (Figure 1d). The interwall spacing was 0.34 nm and the number of graphene walls was approximately 161. The tube length was 4.8 μm. Observing the morphology, we can see a tubular structure with bamboo-like compartments and amorphous carbon deposits around the tube (see supplementary Figure S4). The presence of the catalyst particle at the top of the tube suggests tip-growth [38].

**Figure 2:**
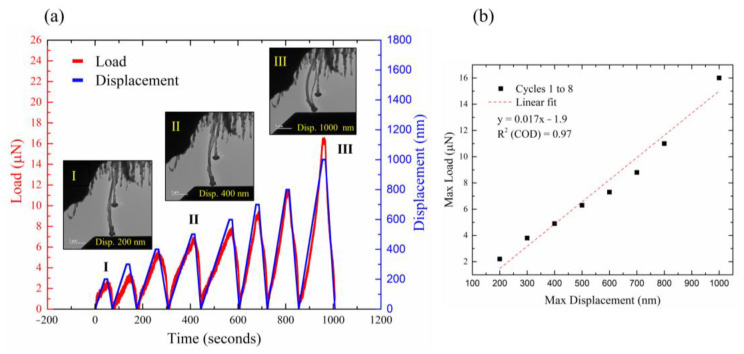
In situ compression tests of a single MWCNT. (**a**) Load–time–displacement curves of eight consecutive compression cycles, each with increased displacement. Inset: corresponding TEM images of selected cycles at the maximum displacement (cycles 1 (I), 4 (II), and 8 (III)); bright-field mode, 200 kV. (**b**) Linear fitting of the maximum load–maximum displacement curve of the same eight cycles, demonstrating the elastic response of the CNT.

The in situ TEM experiments clearly showed the axial buckling process of the individual MWCNT and demonstrated its elastic behavior throughout numerous cycles. Figure 2a illustrates the load (red) and displacement (blue) vs. time graph featuring eight consecutive compression cycles of an individual MWCNT, each with increased maximum displacement. TEM images of the compressed tube at maximum displacement, for three representative cycles (cycles 1, 4, and 8), are shown in the insets of Figure 2a. Each cycle shows an increase in the maximum load and the bending angle of the tube. The maximum displacement can be linearly approximated to the maximum load of each cycle (Figure 2b), demonstrating the elastic behavior of the tube across large displacements.

**Figure 3:**
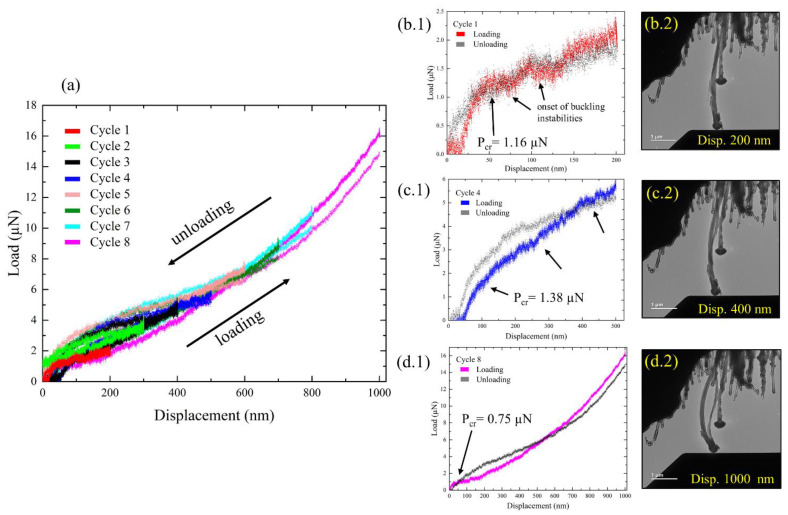
(**a**) Load–displacement curves of eight consecutive compression cycles under axial loading and relaxation. The graph shows increasing displacements and loads for each cycle with a hysteresis. The critical load for buckling (*P_cr_*) is identified in the load–displacement curves and was used to calculate the modulus via Euler’s column formula. (**b**–**d**) Snapshots and the corresponding F–D curves of individual cycles, 1 (**b**), 4 (**c**) and 8 (**d**). The curves show the critical point of buckling and ‘pop-in’ events (marked with black arrows), which reveal the onset of buckling instabilities or failure of the inner graphene walls. The *P_cr_* values of cycles 1, 4, and 8 are 1.16 µN, 1.38 µN, and 0.75 µN, respectively. TEM images were gathered in bright-field mode at 200 kV.

Load–displacement (F–D) curves of the eight cycles are presented in Figure 3a. The experiments began with the diamond tip approaching the tube from the bottom, while slightly negative forces were observed due to electrostatic attraction forces. As the tube underwent compression, the force initially increased linearly with the displacement, indicating the elastic region. When the critical load for buckling was reached, the linear rise transitioned to a force plateau (Figure 3b–d, *P_cr_* in the F–D curves). At that point the tube failed by buckling and started to deflect laterally (bend), as can be seen in the TEM stills (Figure 3(b2,c2,d2)). The *P_cr_* values of cycles 1, 4, and 8 are 1.16 µN, 1.38 µN, and 0.75 µN, respectively. In the post-buckling regime, the F–D curves exhibited a reduction in stiffness (decreased slope) due to lower rigidity in bending [24,25,44], and the system deviated from the ideal linearly elastic response of the pre-buckling regime. This nonlinear response in thick MWCNTs is dictated by the nonlinear mechanics of rippling, exhibiting a bending modulus that increases with deformation [24]. Additionally, ‘pop-in’ events (sudden displacement bursts) are observed in the F–D curves (Figure 3b–d, marked in arrows in the F–D curves) and can be directly correlated to the onset of deformation seen in the TEM (deflection of the tube as was seen in the TEM, Figure S5). These ‘pop-in’ events indicate the movement of inherent defects in the tube and the onset of buckling instabilities. Such defects may include atomic vacancies, Stone–Wales defects, and amorphous regions [45,46,47]. Drops in load can also be attributed to a failure of the inner graphene walls, as will be discussed later. The F–D curves also show a reverse hysteresis behavior, where the force of the unloading segment is, at times, higher than the loading one. This is ascribed to the backward movement of the transducer at large displacements, which results in higher stiffness [48].

Remarkably, even under such large-scale deformation, the compressed nanotube did not suffer catastrophic damage and recovered to its original geometry once the load was released. The structural resilience of CNTs can be attributed to the large in-plane rigidity of graphene sheets, as well as their low rigidity in bending [24,44], and their hollow, large aspect-ratio geometry. In addition, CVD-grown MWCNTs contain many types of defects [47], which may allow them to accommodate high strains [16,25,47,49]. The defects in the tube are arranged in an incoherent fashion such that they separate under tensile stress, and slide reversibly under compressive stress [49].

**Figure 4:**
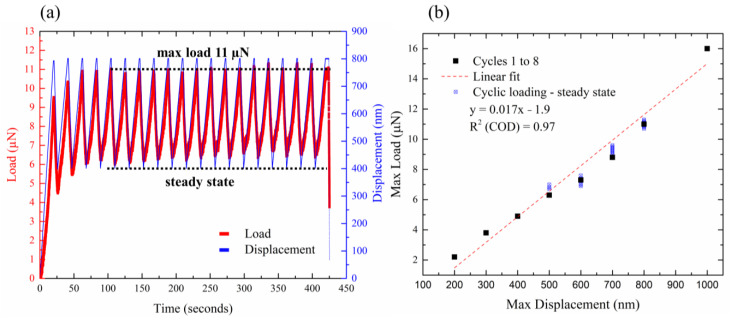
(**a**) Load–time–displacement curves of a representative cyclic compression loading experiment, consisting of 20 deformation cycles to a displacement of 800 nm and then retracting to 400 nm. The loading curve shows an increase in values in the initial four cycles followed by a steady state, where the maximum and minimum loads hardly change between the cycles. (**b**) Maximum load vs. max displacement curve—comparison between the maximum load values in the steady state to the maximum load values in the initial eight cycles of the previous experiment. The blue circles represent the cyclic loading steady state, and the black rectangles represent the eight cycles from Figure 2b. The CNT mechanical response is highly reproducible.

The MWCNT did not fail after the initial eight cycles and was further analyzed in cyclic loading to investigate its durability to mechanical compression. The tube was subjected to 60 more cycles, totaling 68 cycles. Figure 4a presents the final cyclic compression loading experiment, consisting of 20 deformation cycles to a displacement of 800 nm and then retracting to 400 nm. The loading curve shows an increase in maximum value in the initial four cycles, then reaching a steady state where the maximum and minimum loads do not change between the cycles. It may suggest transformation of the inherent defects in the tube by their rearrangement in the initial stages of the experiment. Once the defects are rearranged, the tube is mechanically stable and, thus, a steady state is reached. We then compared the maximum load values in the steady state to the maximum load values in the initial eight cycles of the previous experiment (Figure 2b). The comparison is shown in Figure 4b, with the blue circles representing the cyclic loading steady state and the black rectangles representing the initial eight cycles from Figure 2b. The graphs show that even after numerous loading cycles, the force feedback is almost identical. The experiments demonstrate the ability of the tube to withstand repeated deformations, as well as its elastic response under compression. This is a crucial requirement for flexible electronic devices, as they need to maintain their performance over multiple iterations.

**Figure 5:**
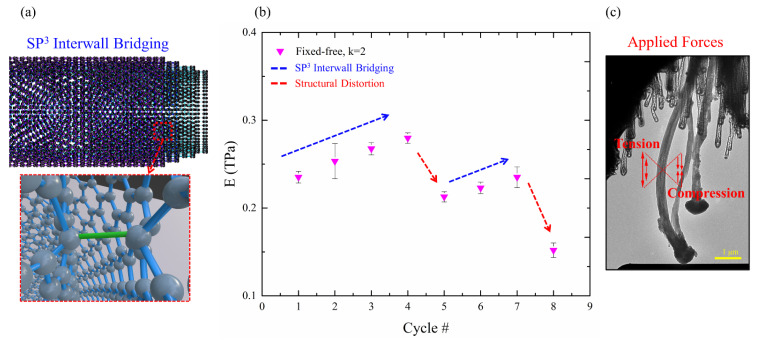
(**a**) Illustration of MWCNT with interwall *sp*^3^ bonding and local distortion in the CNT structure. (**b**) Young’s modulus values per cycle, calculated via Euler’s column formula. The results reveal two competing effects which influenced the tube’s stiffness—interwall *sp*^3^ bridging (blue arrows) and structural distortion (red arrows). The mechanical stiffening may be explained by the creation of interwall *sp*^3^ bonding via electron irradiation which strengthens the bonding of interior walls. The decrease in modulus is attributed to structural distortion caused by the out-of-plane and weaker *sp*^3^ hybridization deforming the layers themselves, making them more susceptible to plastic deformation at higher stresses. (**c**) The bent nanotube underwent repeatable tensile and compressive forces, marked by the arrows, which in time distorted its inner structure and reduced its stiffness.

To validate this experimental methodology, we characterized the stiffness properties of the tube after each cycle and compared those to Young’s moduli of MWCNT found in the literature. By applying Euler’s column formula for the buckling load *P_critical_ = π*^2^*EI/L_e_*^2^, we can estimate Young’s modulus, *E* [17,22]. In this formula, *I* is the cross-sectional moment of inertia of the MWCNT, defined as *I = π* (*d_o_*⁴ − *d_i_*⁴)/64, where *d_o_* and *d_i_* are the outer and inner diameters of the tube, respectively. The effective length of the nanotube is expressed by *L_e_ = KL*, where *L* is the actual length and *K* is the effective-length factor accounting for the end conditions. Based on the end conditions of the experiment, *K* values and, thus, the calculated modulus can vary. We therefore determined that the most suitable end condition for our experiment is a nanocolumn fixed at the top and free at the base (‘fixed–free’), as the tube is strongly attached to the silicon substrate but is free to slide on the indenter’s surface. These conditions set *K* = 2 and *P_critical_ = π*^2^*EI/4L*^2^. The modulus values for each cycle, as estimated through the critical buckling load seen in the F–D curves (*P_cr_* in Figure 3(b1,c1,d1)), are presented in Figure 5b. Notably, the modulus values for the MWCNT calculated with the fixed–free condition, ranging from 0.15 to 0.28 TPa, are in good agreement with the lower range of the values reported in the literature [16,17,19,20,25,35,50], thus validating the experimental methodology. It is expected that the CVD-grown CNT exhibits lower stiffness than tubes grown with the arc-discharge method, due to a lower degree of crystallinity [25,51]. Furthermore, the compartmentalized bamboo-like structure of our tube introduces discontinuities into the layer structure, which act as sites of mechanical weakening [35]. 

However, these values might be a slight under-estimation, considering two more factors. First, the tube was not perfectly straight in the beginning of the experiment. Second, the catalyst particle was not in full contact with the indenter and rotated towards the direction of bending. As a result of these two factors, the mechanical testing was not entirely uniaxial, but rather included bending moments that reduced the ability of the tube to carry load, reducing the critical force. The true modulus should therefore be higher—closer to the mid-range stated in the literature. 

**Figure 6:**
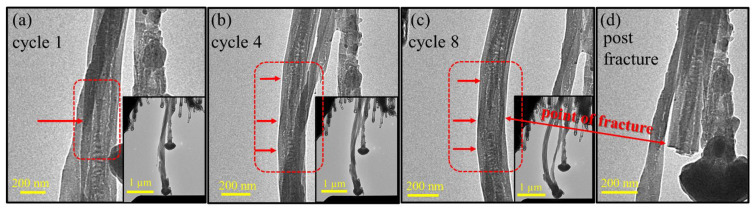
Fracture evolution through cyclic loading. Four TEM snapshots of the bent tube in cycles 1 (**a**), 4 (**b**), 8 (**c**), and post-fracture (**d**). Bright-field mode, 200 kV. (**a**) After cycle 1 a minor defect appeared in the inner void; (**b**) in cycle 4 more defects are observed in the tensile region, marked by the red arrows; (**c**) cycle 8 shows further structural distortion, pointing to ruptures in the tensile region and perhaps a collapse in the inner void. A wave contour is also seen on the compressive side; (**d**) the post-fracture image indicates the location where the fracture evolved, marked by arrows. The near-planar fracture indicates catastrophic brittle behavior.

In Figure 5 we see a dynamic interplay between two competing effects related to continuous exposure to electron beam irradiation and mechanical forces. During experiments, one intermittently dominated over the other, causing opposite outcomes. The electron irradiation induced *sp*^3^ interwall bridging between the graphene layers (Figure 5a). This increased the interlayer stiffness while reducing the in-plane stiffness of the discrete graphene layers. Additionally, the applied tension and compression forces induced local plastic deformations within the tube, as is shown in the fracture analysis (Figure 6). 

The observed stiffening effect, between cycles 1 to 4 and 5 to 7 (Figure 5b, indicated by the blue arrows), is a possible outcome of irradiation annealing caused by the high-voltage electron beam used in the TEM, as the used energy (200 KeV) is known to damage graphitic structures [52]. The irradiation leads to a movement of defects and the creation of *sp*^3^ interwall bridging [23,53,54,55] via heating the sample through energy absorption, and rupturing bonds through electron excitations. Furthermore, high-energy particles can transfer momentum to nuclei, displacing atoms to the interstitial lattice site (‘knock-on’). Such conditions can switch the C–C bond configuration, from in-plane *sp*^2^ into a tetrahedral *sp*^3^ C–C bond, between two adjacent layers (Figure 5a), and vice versa. Normally, the van-der-Waals interactions between the walls of the CNT allow them to slide against each other, but as irradiation increases the formation of covalent bonds between the walls, this substantially increases the shear resistance to sliding, which increases the shear modulus [53,56,57]. The presence of *sp*^3^ bonds also improves the buckling resistance to axial loading by facilitating mechanical participation of the inner walls in the MWCNT, allowing the transfer of load to the inner shells [53,55,57]. Simulations have shown that the modulus can be increased by 25% by *sp*^3^ bridging [58], which fits well with the 19% increase in modulus value observed between cycles 1 to 4 (Figure 5b). Additionally, a high density of *sp*^3^ bonding results in high-stress transfer to the neighboring walls, allowing them to share the load and causing a near-planar fracture [55], as we observed in our experiment (Figure 6d). Considering the length of exposure time to the electron beam during the video recording of the in situ mechanical tests (17 min), our findings are consistent with the electron irradiation stiffening mechanism.

In contrast, after cycles 4 and 7 we observed a sharp decrease in the Young’s modulus (Figure 5b, red arrows). This loss of stiffness can be explained by a few phenomena. Firstly, the effects of electron irradiation on C–C hybridization, where in-plane *sp*^2^ double bonds are converted into weaker out-of-plane *sp*^3^ single bonds, result in a reduction of the in-plane stiffness of the discrete graphene layers. Furthermore, the *sp*^3^ configuration distorts the in-plane layered structure and may act as a weakening point. Secondly, this inner distortion was enhanced by repeated deformation, while the external shape of the tube recovered after the load release. The graphitic layers of the nanotube experienced significant stretching on the outer side of the bending region and significant compression on the inner side (Figure 5c). At later cycles, (cycles 4 to 8), the larger displacements increased the mechanical loads, which were then more likely to cause plastic deformations within the tube, as can be seen in Figure 6. The onset of structural failure can be identified by sudden drops in the force in the mechanical data (Figure 3(b1,c1), marked by black arrows), which may imply that graphene sheets failed in the tensile region. The correlating TEM images indeed reveal points of structural distortion in the tensile region and in the inner columnar void of the tube (Figure 6b, marked with the red arrows), which started to be seen in cycle 4. In cycle 8 (Figure 6c), ruptures were visible in the tensile region and wave contours were seen on the compressive side. These structural distortions can explain the large drops in the modulus values between cycles 4 and 5 and between cycles 7 and 8. Since we used medium magnification in the TEM to view the entire tube, we could not directly observe the broken graphene layers nor the ripples. However, the structural distortion was clearly visible (Figure 6).

After 68 cycles of repeated elastic deformation, the nanotube failed due to fatigue (Figure 6d). The breaking process of the MWCNT was too rapid for the TEM camera to capture. However, its mechanism can possibly be inferred from the mechanical and imaging results. The fatigue of the nanotube occurred due to the distortion of atomic layers in the bent region, manifesting as ripples of the layers on the compressed side, and the breakage of the layers on the tensile side. The resulting fracture mode corresponds to catastrophic brittle failure due to *sp*^3^ crosslinking, as explained earlier. Comparison between the images of cycle 8 and post-fracture indicated the location of fracture evolution (Figure 6c,d, ‘point of fracture’). The images point to the middle of the tube, where the most stress was concentrated, suggesting a collapse of the columnar void along the central axis.

## 4. Conclusions

To summarize, the long-term durability of an individual thick MWCNT to axial compression was investigated utilizing an in situ TEM compression test. The in situ TEM investigation demonstrated the ability of the thick tube to withstand repeated deformation, as well as its elastic response under compression. The tube buckled, yet did not develop more severe plastic deformation such as kinks, allowing it to return to its original geometry. This is a crucial requirement for flexible electronic devices, as they need to maintain their performance over multiple iterations. The buckling behavior was characterized via F–D curves and morphological images, with the tube exhibiting an average critical force for buckling of 1.15 μN. The Young’s modulus was calculated after each cycle using Euler’s column formula, to a range of 0.15 to 0.28 TPa. Based on the moduli values observed, we concluded that the tube structure was distorted by electron beam irradiation and local plastic deformations. Furthermore, the morphological analysis showed the fracture evolution through cyclic loading, pointing to ruptures in the tensile region and a collapse in the inner void. Even though these structural distortions were evident after cycle 4, the tube endured many more loading–unloading cycles (68 in total). In this case, it may indicate that even after the fracture of some layers, there were enough layers in the thick tube to compensate for this loss and still provide adequate compression resistance. Additionally, the cross-links between the inner and outer walls of the MWCNT provided enhancement of interwall strain transfer which also contributed to higher durability. It is important to note that the electron irradiation in the TEM altered the intrinsic properties of the tube, which is a downside of the methodology. It is possible to minimize the irradiation effects without compromising the imaging conditions, by reducing the acceleration voltages of the TEM (e.g., 80 kV) and using aberration correctors. The structure–property relations, studied using in situ TEM, can be used to control CNTs’ properties effectively. By employing the acquired knowledge and controlling the synthesis process, it is possible to produce certain types of CNTs that have desirable properties for each application. Our study exemplifies that when high stiffness is not a priority, but durability and flexibility are needed, thick PECVD-grown tubes can be a better choice.

## Data Availability

The data presented in this study are available upon request from the corresponding author.

## Supplementary Materials

The following supporting information can be downloaded at: https://www.mdpi.com/article/10.3390/nano13020301/s1, Figure S1: Fabrication process of wedge substrates; Figure S2: SEM images of silicon sedge substrates; Figure S3: SEM images of the grown VACNTs; Figure S4: TEM image of the targeted VACNT prior to mechanical testing; Figure S5: Validation of the critical force for buckling. Video S1: In situ TEM compression experiment-cycle 8 (1000 nm maximum displacement). References [40,59] are cited in the supplementary materials.
