# Supplementary material for: Cyclic Buckling Characterization of an Individual MWCNT Using Quantitative In Situ TEM Axial Compression
Source: Nanomaterials (Basel). 2023 Jan 11;13(2):301. doi: 10.3390/nano13020301 (PMC9865386; doi:10.3390/nano13020301)
Supplement: Supplementary file 1 [file nanomaterials-13-00301-s001.zip › nanomaterials-2138725-Supplementary Figures.pdf]

Supplementary Materials

Cyclic Buckling Characterization of an Individual MWCNT using Quantitative In Situ TEM Axial Compression

1. Fabrication process of VACNTs specimens for compression

1.1 Fabrication of Wedge Substrates

A lithographically prepared substrate was designed and fabricated to meet the sample criteria for in situ nanoindentation samples. The process is inspired by the work of Andrew Minor [1]. The wedge structure was designed to be long, narrow, and tall to prevent shadowing from the substrates in the transmission electron microscope (TEM). The fabrication process is described thoroughly and it includes photolithography patterning of a silicon nitride wafer, Reactive Ion Etching (RIE), and a wet etching in KOH solution. The silicon substrates can be produced in large quantities (hundreds of chips per wafer) and with consistent quality.

Figure S1 illustrates the microfabrication process consisting of four steps. In the first step, a 4′′ silicon nitride wafer (P-type, 200 nm nitride layer thickness, <100> crystal orientation, University Wafer Inc, Boston, USA) is spin-coated with a thin adhesive layer (HDMS, MicroChemicals GmbH, Germany) and a positive photoresist (AZ-1505, MicroChemicals GmbH, Germany). The photoresist is "soft-baked" on a hot plate at 115°C for 1.5 minutes. The second step is a photolithography process. The mask was designed using CleWin software (CleWin 4, WieWeb, Twente, Netherlands). The quartz mask was fabricated using Heidelberg Direct laser writing lithography system (DWL 66fs, Heidelberg Instruments, Heidelberg, Germany). The wafer and the mask with the wedge substrate design are aligned with a mask aligner (Karl Suss MA6, Süss Microtec Se, Garching, Germany), followed by applying ultraviolet light. After the exposure to UV, the positive photoresist is developed (AZ-726, MicroChemicals

GmbH, Germany) and the exposed area is washed away, revealing the mask pattern in the photoresist. The third step is selective etching of the nitride layer at the surface by dry etching in RIE (Oerlikon 790 Reactive Ion Etcher, OC Oerlikon, Pfäffikon, Switzerland). The nitride layer is etched everywhere by an etchant gas (CHF_3 200 sccm, etch rate 100 nm/5 minutes), except for under the lines that are covered with the photoresist. At this point, the pattern is marked by the nitride layer over the silicon, with a width of roughly 8 microns where the final ridge will be located.

The fourth step is a wet etching of silicon in potassium hydroxide (KOH). It has been shown that KOH etching of silicon is extremely anisotropic, etching the {111} plane at a much slower rate than that of the {100} and {110} planes. Using KOH to etch a mask line on a wafer oriented at <100>, for example, results in a ridge structure whose sidewalls are the [111] planes oriented 54.7° from the original surface. The substrate with a nitride line 8 μm in width was immersed in a heated solution of KOH (30%) at 80°C for 20 minutes. The silicon is etched under the KOH at different rates according to the orientation of the silicon crystal, while the silicon nitride layer remains unetched. The height and width of the ridge are controlled by the etching time. Figure S2 shows the silicon wedge substrate after 20 minutes of etching, yielding a ridge 30 microns tall and 5 microns wide.

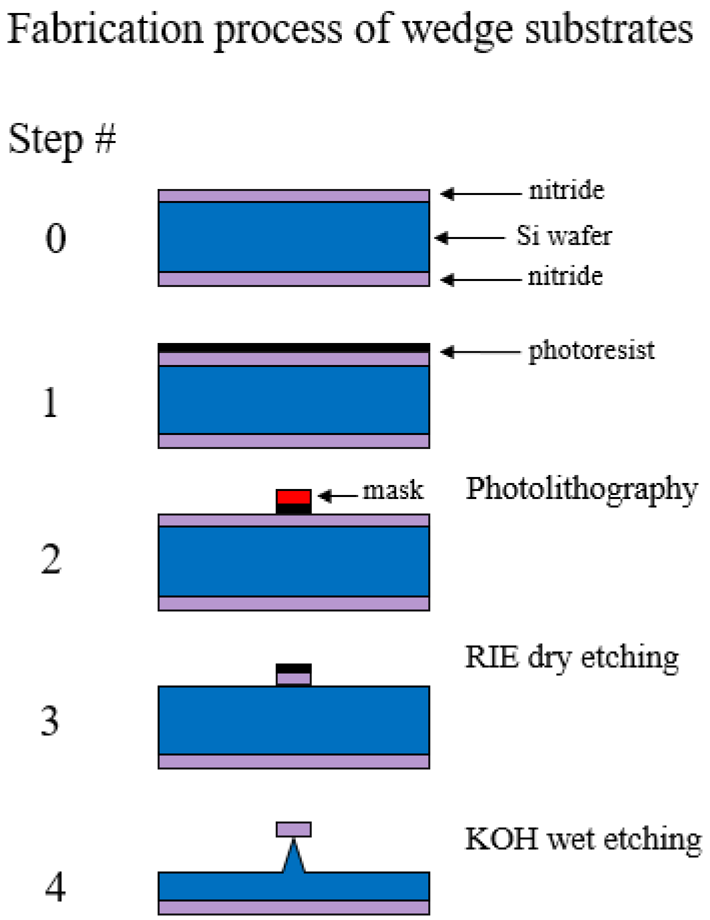

Figure S1. An illustration depicting the fabrication steps used to produce the silicon wedge substrates, viewed in cross-section. (0) A standard P-type <100> silicon wafer with a 200 nm nitride top layer. (1) The wafer is coated with a thin layer of positive photoresist using spin coating. (2) The wafer is inserted into the mask aligner for photolithography. The positive photoresist is exposed to UV light, leaving the masked pattern. (3) Dry etching with RIE to remove the nitride layer except under the photoresist. (4) Wet etching with KOH is used to anisotropically etch the silicon, resulting in wedge structures. The nitride layer acts as a hard mask against the KOH etchant.

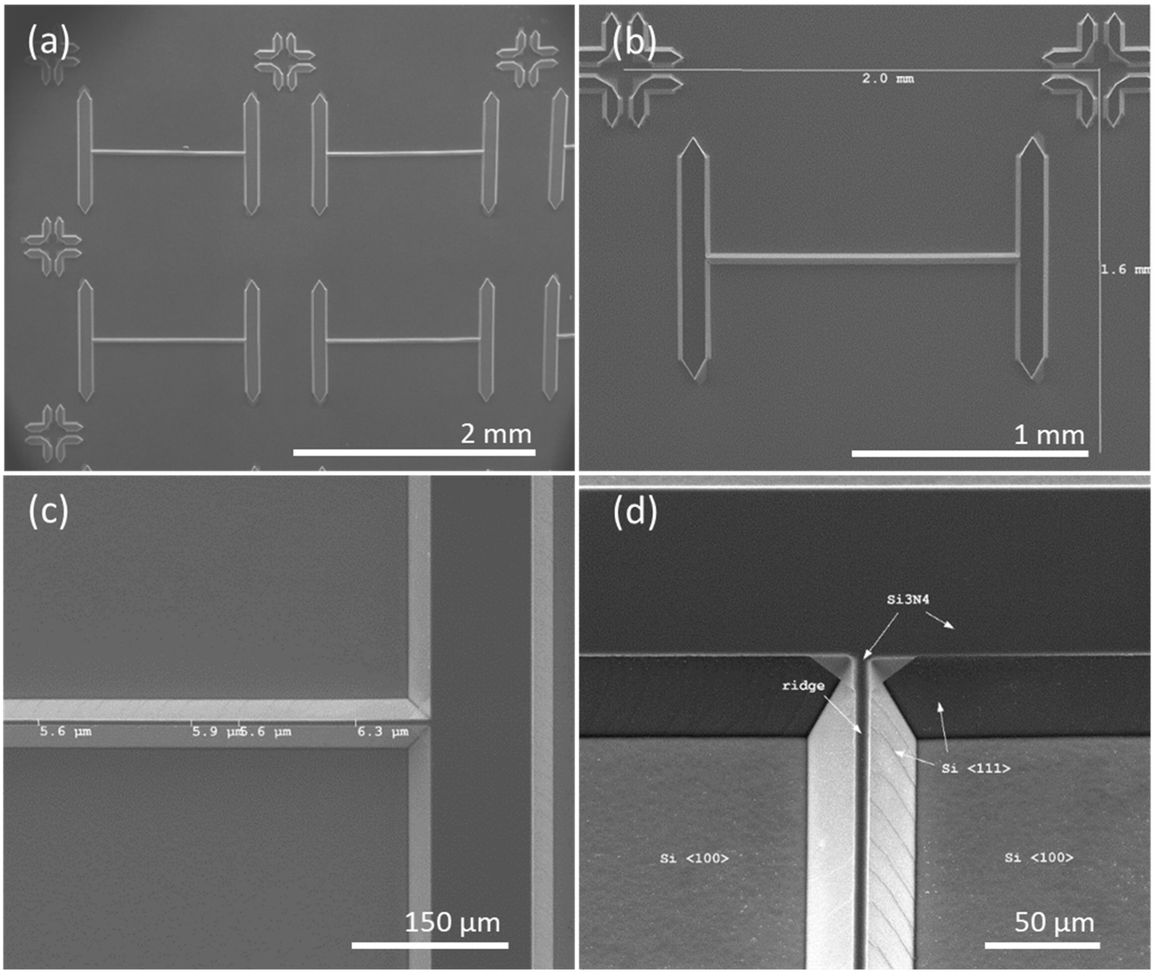

Figure S2. Si wedge substrate after anisotropic etching with KOH 30% solution at 80°C for 20 minutes. (a) wedge substrates array; (b) single substrate in dimensions of 2.0 mm length on 1.6 mm width; (c) the ridge is 6 microns wide; (d) anisotropic etching of KOH creates a V-profile at 54.7° between the <100> and <111> planes.

1.2 VACNTs growth by Plasma-Enhanced Chemical Vapor Deposition

Vertically Aligned MWCNTs were grown on a specially designed silicon wedge substrate by DC/RF Plasma-Enhanced Chemical Vapor Deposition (PECVD, Black Magic 2, Aixtron, Germany) [2]. Initially, the silicon substrates were coated with a 3 nm thick nickel catalyst by an e-beam evaporator (TFDS-680, VST, Kiriat Arie, Israel). The substrates were then placed in the PECVD system on a graphite heater and the chamber was pumped down to a base pressure of 0.2 mbar. The sample was heated to 750°C under a nitrogen atmosphere (200 sccm) at 100 °C/min and was left to anneal for 15 minutes. Subsequently, the temperature was lowered to 700°C and

the N_2 flow was stopped. During the growth stage, a mixture of 20:80 sccm C_2H_2:NH_3 was used as feedstock and DC-plasma was ignited at 550 V under 4 mbar for an hour. The C_2H_2 gas is used as a carbon source and NH_3 is used as a reducing agent. After the CNTs growth, the samples and the chamber were cooled down to room temperature for 1 hour in a nitrogen atmosphere (200 sccm). Figure S3 presents SEM images of the grown VACNTs on top of the silicon ridge.

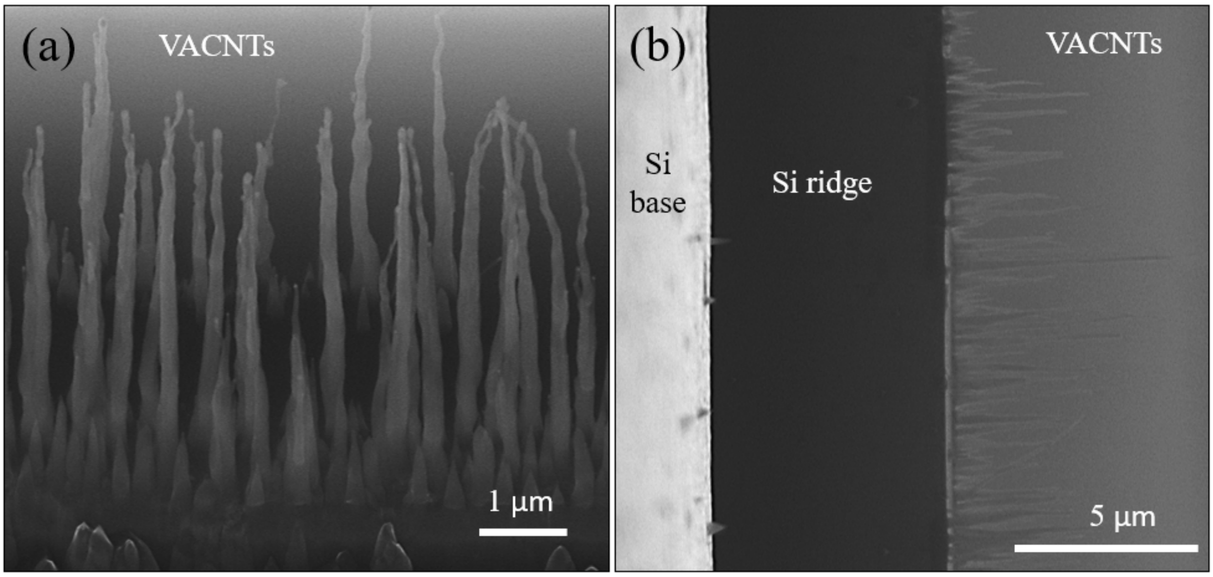

Figure S3. SEM images of the grown VACNTs on the top of the silicon ridge. (a) 30° tilt-view of the ridge containing VACNTs; (b) 90° tilt-view of the VACNTs, demonstrating the orientation of the silicon substrate as in the TEM.

2. Structure and morphology analysis of targeted VACNT

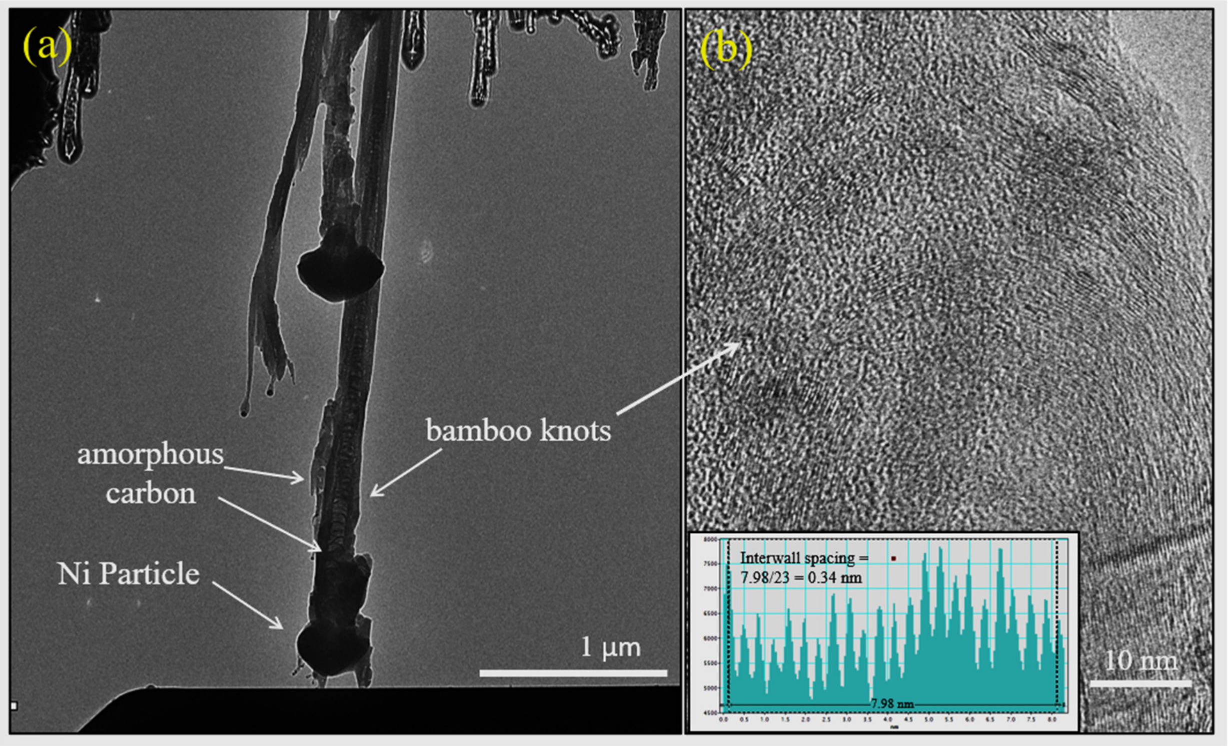

Figure S4. (a) A full TEM image of the targeted VACNT prior to the mechanical testing reveals the structure of the CNT; (b) high-magnification image of the graphitic layers, revealing the bamboo compartments and interwall spacing of 0.34 nm, measured with the histogram profile (inset). The image was taken at 200 kV, bright-field mode.

Figure S4a presents an enlarged TEM image of the targeted VACNT as in figure 1c in the manuscript. The image shows the morphology of the tube, revealing a tubular structure with bamboo-like compartments and amorphous carbon deposits around the tube. The position of the nickel particle at the top of the tube suggests that a tip growth occurs. Figure S4b reveals the graphitic layer, their interwall spacing of 0.34 nm, and the bamboo compartments. The interwall spacing is determined using the histogram profile and is calculated by averaging the distance between 23 peaks (representing the walls) - a measured length of 7.98 nm divided by 23 (the number of peaks).

3. Validation of the critical force for buckling

One of the benefits of in situ TEM method is that the mechanical data is supplemented by the morphological imaging. This was useful in validating the critical load for buckling which is accurately determined by calculating the tangent point between two linear fits of the pre- and post-buckling regions, as mentioned in the manuscript. It was also validated with the synchronized video of the F-D curves and the real-time imaging, by identifying the point at which lateral deflection begins, as can be seen in Figure S5.

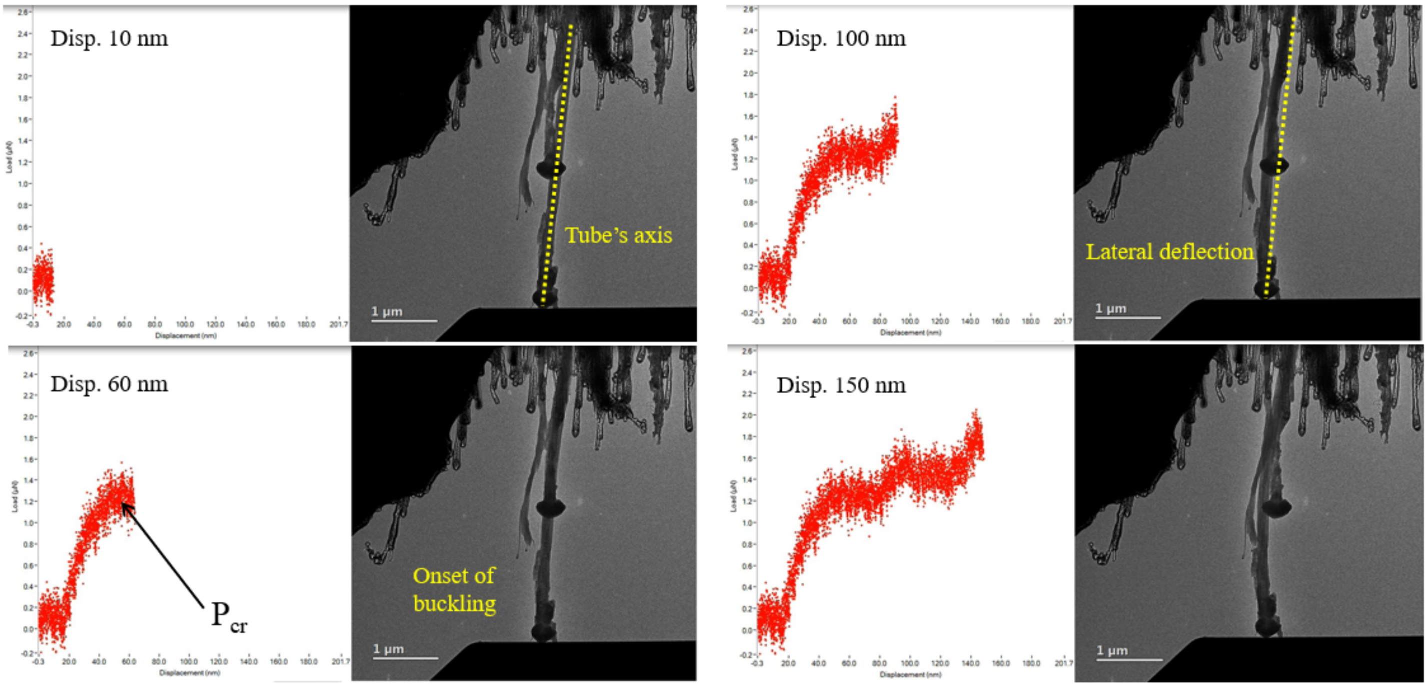

Figure S5. Validation of the critical load for buckling with the synchronized video – example of cycle 1. Four snapshots of the cycle 1 experiment as seen in the TriboScan software, at increasing displacements. The Pcr is identified in the F-D curve, seen at a displacement of 60 nm, and then it's confirmed in the imaging by detecting the onset of lateral deflection.

References

1. Andrew, M.; Minor, A.M. In Situ Nanoindentation in a Transmission Electron Microscope. *Dr. Thesis* **2002**.

2. Harpak, N.; Davidi, G.; Melamed, Y.; Cohen, A.; Patolsky, F. Self-Catalyzed Vertically Aligned Carbon Nanotube-Silicon Core-Shell Array for Highly Stable, High-Capacity Lithium-Ion Batteries. *Langmuir* **2020**, *36*, 889–896, doi:10.1021/acs.langmuir.9b03424.
